# Sponsors’ participation in conduct and reporting of industry trials: a descriptive study

**DOI:** 10.1186/1745-6215-13-146

**Published:** 2012-08-24

**Authors:** Andreas Lundh, Lasse T Krogsbøll, Peter C Gøtzsche

**Affiliations:** 1 The Nordic Cochrane Centre, Rigshospitalet, Dept., 7811, Blegdamsvej 9, DK-2100, Copenhagen, Denmark; 2 Institute of Medicine and Surgery, Faculty of Health Sciences, University of Copenhagen, Blegdamsvej 3b, DK-2200, Copenhagen, Denmark

**Keywords:** Randomised trials, Industry sponsorship, Academic authors, Trial protocols

## Abstract

**Background:**

Bias in industry-sponsored trials is common and the interpretation of the results can be particularly distorted in favour of the sponsor’s product. We investigated sponsors’ involvement in the conduct and reporting of industry-sponsored trials.

**Methods:**

We included all industry-sponsored trials published in *The Lancet* in 2008 and 2009 and corresponding trial protocols provided by *The Lancet*. For each protocol and publication, we extracted information on trial conduct and reporting.

**Results:**

We identified 169 publications of randomised trials and included 69 (41%) that were industry-sponsored, and 12 (7%) industry-funded but seemingly independently conducted as a subsample. Entry of data into the study database was done independently by academic authors without the involvement of the sponsor or a contract research organisation in one of the 69 trials. Two trials had independent data analysis and one independent reporting of results. In 11 of the trials, there was a discrepancy between the information in the protocols and papers concerning who analysed the data. In four of the 12 seemingly independent trials, the protocol described sponsors’ involvement in writing the report while the published paper explicitly stated that the sponsor was not involved.

**Conclusions:**

The sponsors are usually involved in the analysis and reporting of results in industry-sponsored trials, but their exact role is not always clear from the published papers. Journals should require more transparent reporting of the sponsors’ role in crucial elements such as data processing, statistical analysis and writing of the manuscript and should consider requiring access to trial protocols, independent data analysis and submission of the raw data.

## Background

The drug and device industries have a major impact on the research agenda. They funded 58% of US biomedical research in 2007 [1], and 56% of trials published in high-impact medical journals in 2005 and 2006 had industry funding; for *New England Journal of Medicine* it was 78% [2]. The involvement of the company in industry-sponsored trials varies from no involvement, besides the free provision of drugs, to running the whole trial and publishing the results without the involvement of academic authors.

Industry-sponsored trials usually favour the company’s product [3,4]. This may happen through biases in study design, choice of comparators or selective reporting of favourable outcomes [5,6]. Some journals therefore require that the involvement of the sponsor is stated in the published article. *JAMA* goes further and requires independent data analysis by academic authors [7].

Many industry-sponsored trials are coordinated by seemingly independent steering committees. However, this may not prevent sponsor influence, as academic authors often have constraints on publication rights [8,9], the sponsor often owns the data [9,10], ghost authorship is common [11], and academic authors may have industry ties [12].

We have previously reported the results from a cohort of trials published in *The Lancet* in 2008 and 2009 [10]. We found that academic authors involved in industry-sponsored trials may have limited access to the raw data, although they all declared in *The Lancet* that they had full access to the data. We report here on the sponsors’ influence on trial conduct and reporting of the results.

## Methods

### Sample

We identified all randomised clinical trials published in *The Lancet* in 2008 and 2009 using the index term ‘randomized controlled trial’ in PubMed. We excluded papers that were not full trial reports (for example, letters and commentaries) or were not part of the planned trials (for example, secondary analyses). We selected all industry-sponsored trials, defined as trials fully funded by a drug or device company and where the sponsor participated in data management or analysis. Trials where part of trial conduct was outsourced to a contract research organization (CRO) were also included. Trials where all elements of trial conduct were managed by independent academic authors (for example, by ’unrestricted’ grants) were analysed separately.

Since July 2002, *The Lancet* has required authors to submit protocols together with the trial report and we retrieved copies of these protocols.

### Information on trial conduct and reporting in protocols and papers

One of us (AL) copied all information from protocols and papers on data management, storage, analysis, and writing of the protocol and manuscript into a pilot-tested data sheet. Two observers (AL, LTK) independently categorized these data into prespecified domains for protocols and papers, and disagreements were resolved by discussion and arbitration when needed by the third observer (PG). We made a final categorization based on data from both protocols and published papers and described discrepancies.

## Results

**Figure 1:**
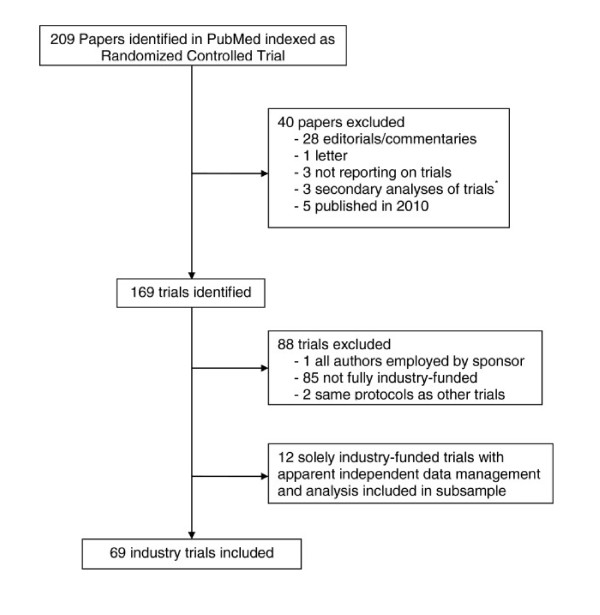
Flow chart showing inclusion of trials.^*^Secondary analysis refers to when the trial data were used in an exploratory fashion. For example, the placebo group was analysed as a separate cohort study to investigate heart rate as a predictor for cardiovascular mortality in a trial of an anti-arrhythmic drug.

We identified 209 papers in PubMed and excluded 40 that were not primary reports of trials published in 2008 and 2009 (Figure 1). We excluded another 85 trials that were not fully funded by the industry, two that had protocols similar to other included trials, and one that had no independent academic authors. Of the remaining 81 trials, we included 69 industry-sponsored trials. The other 12 trials were also fully industry-funded but appeared to have been independently conducted and we therefore analysed them separately.

For seven trials, the full protocols were missing: two were not in *The Lancet’s* database, three were protocol synopses, one was a copy of the information from http://www.clinicaltrials.gov and one only consisted of amendments to the protocol. We received copies of five protocols from the academic authors and the other two from the sponsors.

### Data management

In 49 of the 69 trials (71%), review and verification of information in case report forms (CRFs) were handled by the sponsor or a CRO without involvement of academic authors and only in two trials (3%) by academic authors independently (Table 1).

**Table 1:** Data management and analysis in industry-sponsored trials based on information in protocols and publications

**(n = 69)**	**CRF review and verification**	**Data entry**	**Data storage**	**Data analysis**
**Sponsor**	23 (33%)	32 (46%)	35 (51%)	29 (42%)
**Sponsor and CRO**	18 (26%)	8 (12%)	3 (4%)	6 (9%)
**CRO**	8 (12%)	12 (17%)	6 (9%)	5 (7%)
**Sponsor/CRO and academic authors**	10 (14%)	0 (0%)	0 (0%)	11 (16%)
**Academic authors**	2 (3%)	1 (1%)	1 (1%)	2 (3%)
**Not described**	8 (12%)	16 (23%)	23 (33%)	5 (7%)
**Discrepancy between protocol and paper**	0 (0%)	0 (0%)	1 (1%)	11 (16%)

Percentages do not always add up to 100 due to rounding.

In 52 trials (75%), entry of data into the study database was done by the sponsor or a CRO without involvement of academic authors. In only two of these trials, it was described how data were processed, that is, interpreted for categorisation purposes, and in those two trials, all safety data were processed by sponsor staff. In only one trial (1%) was data entry performed independently by academic authors. However, based only on information in the published paper, it was not possible to tell who entered study data in 50 (72%) of the trials. In 44 trials (64%), the data were stored by the sponsor or a CRO and only in one trial (1%) by academic authors. In one trial (1%), the protocol suggested that academic authors stored the data whereas the paper suggested that the sponsor stored it.

According to 38 trial protocols (55%), the sponsor had access to accumulating data before study termination and according to two (3%) the sponsor had access via membership of the Data and Safety Monitoring Board. In 24 of these 40 trials, the sponsor could stop the trial prematurely for a broad range of reasons or without any constraints at all, in five additional trials it could also be stopped but no criteria were specified, and in the remaining 11 trials it was not described whether the sponsor could stop the trial prematurely.

### Data analysis

In 40 trials (58%), the data were analysed by the sponsor or a CRO without involvement of academic authors and only in two trials (3%) independently by academic authors. In 11 trials (16%) the sponsor or a CRO, and academic authors analysed the data. However, in six of these trials, the sponsor or CRO biostatistician had the primary role and in five, the role was not clear as many authors were listed. In one of these five trials, the protocol named a sponsor-employed study biostatistician who was not mentioned in the paper.

In an additional 11 trials (16%), there were discrepancies between information in protocols and papers. In four, the protocol described analysis by sponsor or CRO alone whereas the published paper described analysis either by academic authors alone or in collaboration with the sponsor. In five trials, there were discrepancies between information in protocols and papers as to whether the sponsor or a CRO did the analysis, and in two the protocol described an independent analysis by academic authors whereas the papers also described involvement of a CRO or the sponsor. Based only on information in the published paper, it was not possible to tell who analysed the data in another 10 (14%) of the trials.

### Publication of the results

In 24 of the 69 trials (35%), the sponsor or a hired CRO was involved in coordinating writing of the manuscript, in 10 (14%) the sponsor was not involved and in 35 (51%) it was not described. In 64 trials (93%), the sponsor had influence over publication of the results through co-authorship or an explicitly stated right to approve, review or comment on the paper (Table 2). In three trials (4%), there were discrepancies between information in protocols and papers: one protocol described sponsor-employed co-authors without this being stated in the paper; in one protocol the sponsor needed to approve the manuscript, but the paper stated that the sponsor was not involved in writing of the report; and in one protocol the sponsor needed to approve the manuscript, but the paper stated that the report was written in consultation with the sponsor.

**Table 2:** Sponsors’ influence on publication of results of industry-sponsored trials based on information in protocols and publications

**(n = 69)**	**Publication of results**
**Sponsor has co-authorship**	56 (81%)
**Sponsor needs to approve manuscript**	3 (4%)
**Sponsor needs to review or comment**	5 (7%)
**No influence**	1 (1%)
**Not described**	1 (1%)
**Discrepancy between protocol and paper**	3 (4%)

Percentages do not add up to 100 due to rounding.

Ten of the protocols (14%) referred to separate agreements (for example, clinical trial agreements or publication agreements) concerning reporting of results or data ownership and five other protocols (7%) stated that such agreements might be issued, overriding statements concerning reporting of results or data ownership in the protocol. None of these agreements had been provided to *The Lancet*. Finally, five protocols explicitly described that the sponsor could publish the results without author approval, but this did not seem to have happened (there were academic authors on all 69 papers).

Medical writing assistance from the sponsor or persons hired by the sponsor was described in 37 of the 69 papers (54%), in seven papers (10%) it seemed no assistance was provided and in 25 (36%) it was not described. Sixty-eight of the 69 protocols (99%) seemed to have been written by the sponsor, for example, by including the company logo, and one protocol contained no information indicating who had written it. In 19 protocols, specific authors were named and 10 of these protocols specified them as authors of the protocol. However, for 14 of these 19 protocols these authors were not mentioned in the publications.

### Independently conducted trials

In eight of the additional 12 trials that appeared to have been conducted independently of the sponsor, the sponsor nevertheless appeared to have written the protocol, could stop the trial early, had issued confidentiality clauses or had influence on writing of the manuscript (Table 3).

**Table 3:** Eight seemingly independent trials with evidence of influence by sponsor

**Trial number**	**Writing of protocol**	**Stopping trial early**	**Author confidentiality clause**	**Writing of manuscript**	
				**According to protocol**	**According to publication**
1	-	-	In protocol	-	Sponsor not involved in writing
2	-	-	-	Sponsor needs to review manuscript	Sponsor not involved in writing
3	Appears written by sponsor	-	-	Sponsor involved in writing	Sponsor not involved in writing
4	-	-	-	Sponsor needs to review manuscript	Sponsor not involved in writing
5	-	Sponsor must be consulted before stopping	In protocol	Sponsor needs to approve manuscript	Sponsor not involved in writing
6	-	-	-	Sponsor needs to review manuscript	Nothing stated about writing
7	-	-	In protocol	-	Sponsor not involved in writing
8	-	Sponsor can stop trial	-	Sponsor allowed comments	Sponsor allowed comments

- Indicates no evidence of influence by sponsor.

## Discussion

Approximately half the trials published in *The Lancet* were fully funded by the industry and most of these had industry involvement in the conduct, analysis and reporting of the results. The sponsor often entered, stored and owned the data, which were rarely analysed independently by the academic authors. Even for the additional 12 trials that appeared to have been conducted independently of the sponsor, the sponsor could stop the trial prematurely in some cases, issued confidentiality clauses, was involved in the reporting of results or appeared to have written the protocol.

Our study describes sponsor involvement in the conduct and reporting of industry-sponsored trials published in a high-impact medical journal. Our access to trial protocols gave us additional information on sponsor involvement not possible to decipher from the published papers alone. A study of cancer trials found that only 18% of the industry-sponsored trials described the sponsors’ role and usually in vague terms [13]. There are some limitations that should be taken into account though. First, we restricted our sample to trials published in a single journal, *The Lancet*, which may limit generalisability. However, *The Lancet’s* access to protocols and editorial resources might indicate that the sponsors’ role is greater for trials published in other journals. Second, despite access to trial protocols, in many cases we could not tell who entered, processed or stored data, and we did not have copies of trial agreements and publication agreements. We find it likely that tasks not described were handled by the sponsor because the protocols in all except one case were most likely written by the sponsor. It might therefore be regarded as implicit that what had been left out would be managed by the sponsor. The role of the sponsor may therefore be even more extensive than our results indicate.

Bias in industry-sponsored trials can be introduced at various levels of data processing, from the information being recorded on CRFs to the data appearing in the published paper. In most cases, the sponsor or a hired CRO was in charge of data entry and while it was rarely described, they probably also processed the data for categorisation purposes.

Processing data is bias-prone. Important data are often omitted from publications or are described in a way favourable for the sponsor. For example, suicidality was coded as ‘emotional lability’, ‘hospital admission’ or ‘lack of effect’ in trials of selective serotonin reuptake inhibitors (SSRIs) [14,15], myocardial infarctions on rofecoxib were omitted in the VIGOR trial [16,17] and on rosiglitazone in the RECORD trial [18,19]. As academic authors were rarely involved in data entry, and as data analysis by academics often did not involve anything more than checking the tabulated data in the clinical study report [10], such practices will most likely not be discovered.

Academic authors were rarely involved in data analysis and only two trials had a completely independent analysis. When data analysis was performed jointly, the sponsor seemed to take the leading role and, for some trials, the role of academic authors in the actual statistical analysis was probably limited, as many authors were named as contributors to data analysis. We find it highly unlikely that many academic authors with a clinical background actually participated in the statistical analysis, as such analyses are time-consuming and require statistical expertise. Based on our previous study [10], such involvement might actually, again, be limited to merely reading the clinical study report.

Data analysis done solely by the sponsor is problematic, as independent analysis may yield different results [20]. In some cases, the data were analysed by CROs, but they are not independent. They are hired by the sponsor, they sometimes have financial interests in the sponsoring companies [21,22], and - like for medical writers - if they do not do a job that satisfies the sponsors’ marketing department, they might go out of business. Furthermore, analysis by academic authors does not ensure independence, as such authors often have financial ties to the industry [12]. Based on their declarations in the paper, in the two trials with independent analysis, the academic authors were paid by the companies for their contribution.

The sponsors’ dominating role in data analysis is not only problematic in relation to selective reporting of positive outcomes and spin of the results [23,24], but also in relation to stopping trials early. If the sponsor has access to accumulating data, as was the case for at least 40 trials, and is allowed to terminate the trials prematurely, this could lead to overestimation of treatment effects [25] and underestimation of harms [26].

Journal editors should consider whether independent statistical analysis by academic authors should be a requirement, as is the case for *JAMA*[7]*.* This policy has had repercussions, as fewer industry-sponsored trials have been published [27]. Such policies might therefore be difficult to implement, as they will likely result in loss of revenue from reprint sales of industry trials [2]. However, this only reinforces the need for independent analyses. Lastly, journals should require copies of protocols and any additional agreements to ensure that access to the data was planned before the trial started. Journals should allocate editorial resources to ensure that what appears in publications corresponds to statements written in protocols, which was not always the case in our study. Such protocols should be written in accordance with evidence-based standards such as the upcoming SPIRIT guidelines [28] and should contain detailed information on authors’ access to data. To ensure that such declarations are more than window dressing [10], journal editors might also consider asking for the raw data as a condition for publication, like *Science* and the *Nature* journals require [23] and preferably make such data available at public websites.

## Conclusions

The sponsors are usually involved in the analysis and reporting of the results in industry-sponsored trials, but their exact role is not always clear from the published papers. Even for fully industry-funded trials that appear to have been conducted independently, the sponsors are also sometimes explicitly involved in the reporting of results. Journals should require more transparent reporting of the sponsors’ role in crucial elements such as data processing, statistical analysis and writing of the manuscript and should consider requiring access to trial protocols, independent data analysis and submission of the raw data.

## Competing interests

The authors declare that they have no competing interests.

## Authors’ contributions

PCG conceived the idea for the study. The protocol was primarily developed by AL, and LTK and PCG contributed. AL identified trials and protocols; AL and LTK extracted data. All authors participated in data analysis and writing of the paper. AL, LTK and PCG are guarantors. All authors had full access to all the data in the study and take responsibility for the integrity of the data and the accuracy of the data analysis. All authors read and approved the final manuscript.

## Ethics

The study was based on protocol data and published data and did not need ethical approval according to the Danish Act on a Biomedical Research Ethics Committee System and the Processing of Biomedical Research Projects.

## Funding source

The study was partly funded by The Health Insurance Foundation and The Danish Council for Independent Research - Medical Sciences, partly by The Nordic Cochrane Centre.

## Role of sponsors

The study was conducted independently of study sponsors. There was no sponsor involvement in the design; collection, analysis, and interpretation of the data; in writing of the report; or in the decision to submit for publication.
